# DISABILITY STATUS, FINANCIAL STRAIN, AND SUBJECTIVE HEALTH AND WELL-BEING FOR PEOPLE WITH LTSS NEEDS IN CALIFORNIA

**DOI:** 10.1093/geroni/igac059.1047

**Published:** 2022-12-20

**Authors:** Lei Chen, Kathryn Kietzman, Fernando Torres-Gil

**Affiliations:** University of California, Los Angeles, Los Angeles, California, United States; University of California, Los Angeles (UCLA), Los Angeles, California, United States; University of California, Los Angeles (UCLA), Los Angeles, California, United States

## Abstract

Many people with needs for Long-Term Services and Supports (LTSS) are vulnerable to financial strain, a chronic economic stressor that may negatively affect a person’s well-being. This study examines the extent to which financial strain mediates the relationship between people’s disability status and subjective health and well-being, controlling for select demographic characteristics. Disability status refers to the intensity of disabilities that people report, including cognitive impairments, and/or difficulties performing activities of daily living and/or instrumental activities of daily living. Financial strain measures the number of challenges that participants incurred during the last year in acquiring food, housing, health care, and other basic needs. We use the first cycle of data (2019-2020) from the California Long-Term Services and Supports (LTSS) survey, merged with select data from the California Health Interview Survey (CHIS) (N = 2,030). Drawing from Pearlin’s Stress Process Model, we use Conditional Process Analysis (CPA) to examine the hypothesized mediation relationships. Findings show that the intensity of disability status has a direct association with self-rated health (c’ = -.2054, p < .0001) and psychological distress (c’ = .7247, p < .0001). Furthermore, financial strain experienced by people with LTSS needs mediates the relationship between their disability status and 1) self-rated health (ab = -.0178, BootCI= -.0285 to -.0082) and 2) psychological distress (ab = 0.19, BootCI= .1323 to .2648). These results have policy and practice implications for national and state programs, such as Medicaid, the Universal Basic Income (UBI) program, and the Master Plan for Aging in California.

